# Supplementary material for: The Diagnostic Values of Peptidoglycan, Lipopolysaccharide, and (1,3)-Beta-D-Glucan in Patients with Suspected Bloodstream Infection: A Single Center, Prospective Study
Source: Diagnostics (Basel). 2022 Jun 14;12(6):1461. doi: 10.3390/diagnostics12061461 (PMC9221811; doi:10.3390/diagnostics12061461)
Supplement: Supplementary file 1 [file diagnostics-12-01461-s001.zip › diagnostics-1746089-Table S1.pdf]

Supplementary table 1 Classification of bacteria in peripheral blood and the detection results of multimarker detection approach for BSI

Gram negative bacteria	Number (number detected)	Proportion in total (detection proportion in total)	Gram positive bacteria	Number (number detected)	Proportion in total (detection proportion in total)	Fungi	Number (number detected)	Proportion in total (detection proportion in total)
Escherichia coli	18(15)	28.6% (23.8%)	*Staphylococcus epidermidis*	6(5)	15.0% (12.5%)	*Candida albicans*	4(2)	50% (25%)
Klebsiella pneumoniae	22(21)	34.9% (33.3%)	*Staphylococcus aureus*	6(6)	15.0% (15.0%)	*Candida tropicalis*	1(0)	12.5% (0)
Klebsiella aerogenes	2(2)	3.2% (3.2%)	*Staphylococcus pettenkoferi*	1(1)	2.5% (2.5%)	*Scedosperium apiospermum*	1(1)	12.5% (12.5%)
Pseudomonas aeruginosa	6(6)	9.5% (9.5%)	*Staphylococcus hominis*	4(1)	10.0% (2.5%)	*Trichosporon asahii*	1(1)	12.5% (12.5%)
Acinetobacter baumannii	6(4)	9.5% (6.4%)	*Enterococcus faecalis*	3(3)	7.5% (7.5%)	*Cryptococcus neoformans*	1(1)	12.5% (12.5%)
Acinetobacter Pitt	1(1)	1.6% (1.6%)	*Enterococcus faecium*	8(8)	20.0% (20.0%)			
Bacteroides thetaiotaomicron	2(2)	3.2% (3.2%)	*Streptococcus mitis*	2(2)	5.0% (5.0%)			
Bacteroides fragilis	1(1)	1.6% (1.6%)	*Streptococcus oralis*	1(1)	2.5% (2.5%)			
Bacteroides ovatus	1(0)	1.6% (1.6%)	*Streptococcus gallolyticus*	1(1)	2.5% (2.5%)			
Salmonella enteritidis	1(1)	1.6% (1.6%)	*Streptococcus constellatus*	1(1)	2.5% (2.5%)			
Fusobacteriumulcerans	1(1)	1.6% (1.6%)	*Streptococcus agalactiae*	1(1)	2.5% (2.5%)			
enotrophomonas maltophil	1(0)	1.6%(0)	*α-streptococcus haemolyticus*	2(1)	5.0% (2.5%)			
Enterobacter amnigenus	1(0)	1.6%(0)	*Bifidobacterium bifidum*	1(0)	2.5% (0)			
			on-fermenting corynebacteriu	1(0)	2.5% (0)			
			Enterococcus raffinosus	1(0)	2.5% (0)			
			Propionibacterium Acnes	1(0)	2.5% (0)			
Total	63(54)	100% (87.3%)	Total	40(31)	100% (77.5%)	Total	8(6)	100% (62.5%)
