# Supplementary material for: The Diagnostic Values of Peptidoglycan, Lipopolysaccharide, and (1,3)-Beta-D-Glucan in Patients with Suspected Bloodstream Infection: A Single Center, Prospective Study
Source: Diagnostics (Basel). 2022 Jun 14;12(6):1461. doi: 10.3390/diagnostics12061461 (PMC9221811; doi:10.3390/diagnostics12061461)
Supplement: Supplementary file 1 [file diagnostics-12-01461-s001.zip › diagnostics-1746089-Table S2.pdf]

Supplementary table 2 Classification of bacteria in sterile body fluids or other sterile tissues and the detection results of mul

Gram negative bacteria	Number (number detected)	Proportion in total (detection proportion in total)	Gram positive bacteria	Number (number detected)	Proportion in total (detection proportion in total)
Escherichia coli	4(2)	23.5% (11.8%)	*Staphylococcus epidermidis*	1(1)	9.1% (9.1%)
Klebsiella pneumoniae	4(2)	23.5% (11.8%)	*taphylococcus haemolyticu*	1(0)	9.1% (0%)
Klebsiella aerogenes	1(0)	5.9% (0%)	*Enterococcus faecium*	5(3)	45.5% (27.3%)
Bacteroides fragilis	1(0)	5.9% (0%)	*Enterococcus faecalis*	1(0)	9.1% (0%)
Acinetobacter baumannii	2(0)	11.8% (0%)	*Bacillus cereus*	1(0)	9.1% (0%)
Enterobacter cloacae	2(1)	11.8% (5.9%)	*Streptococcus constellatus*	2(0)	18.2% (0%)
enotrophomonas maltophil	1(0)	5.9% (0%)			
Citrobacter freundii	1(0)	5.9% (0%)			
Pseudomonas aeruginosa	1(1)	5.9% (0%)			
Total	17(6)	100% (35.3%)	Total	11(4)	100% (36.4%)

timarker detection approach for BSI

Fungi	Number (number detected)	Proportion in total (detection proportion in total)
Candida albicans	3(2)	13.0% (8.7%)
Candida tropicalis	2(0)	8.7% (0%)
Candida glabrata	2(2)	8.7% (8.7%)
Pneumocystis jiroveci	16(16)	69.6% (69.6%)
Total	23(20)	100% (87.0%)
