# Supplementary figures and images for: The Diagnostic Values of Peptidoglycan, Lipopolysaccharide, and (1,3)-Beta-D-Glucan in Patients with Suspected Bloodstream Infection: A Single Center, Prospective Study
Source: Diagnostics (Basel). 2022 Jun 14;12(6):1461. doi: 10.3390/diagnostics12061461 (PMC9221811; doi:10.3390/diagnostics12061461)

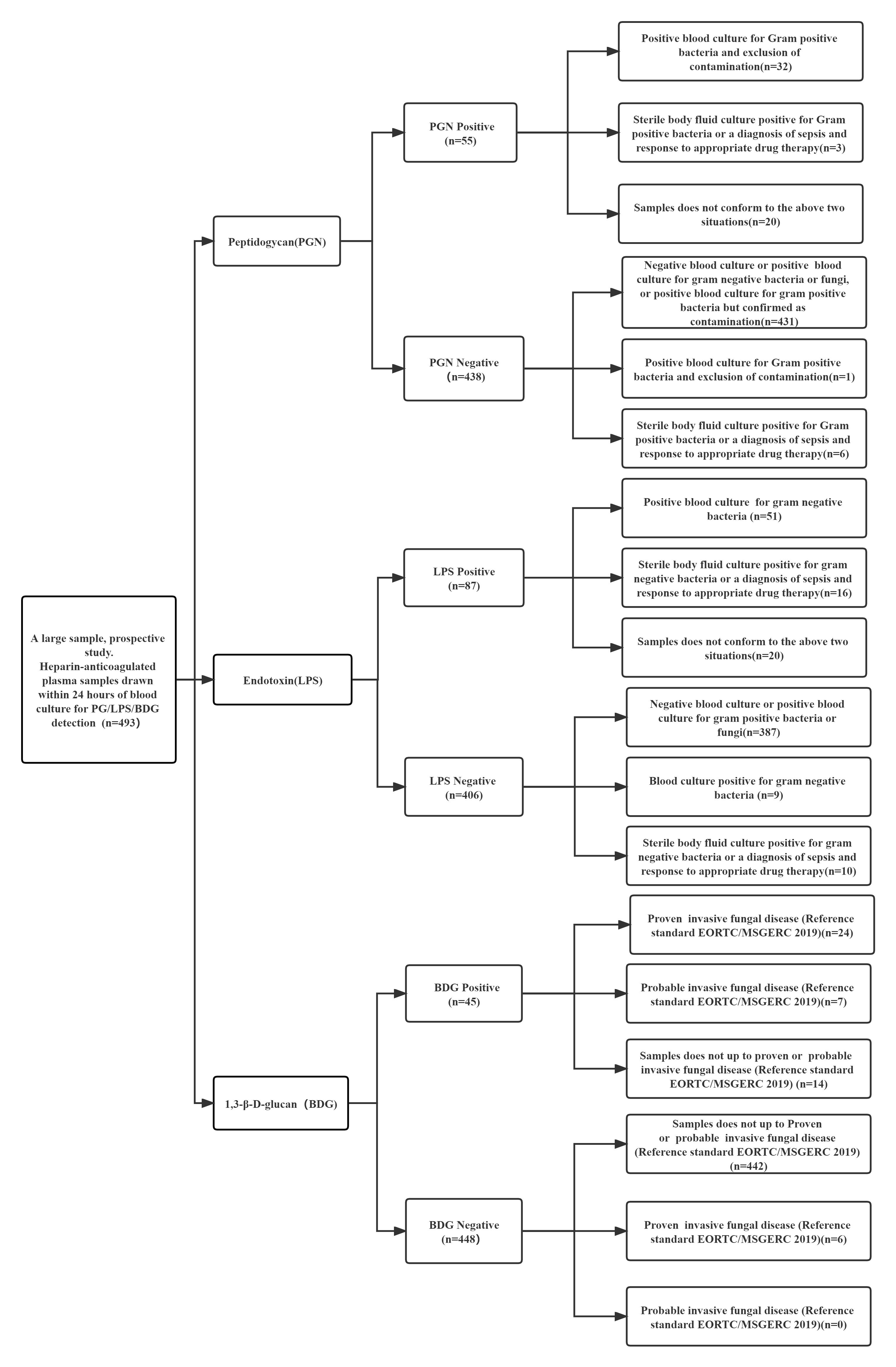

Supplement: Supplementary file 1 [file diagnostics-12-01461-s001.zip › diagnostics-1746089-Figure S1.png]
